# Duloxetine for treatment of male sphincteric incontinence following partial conus medullaris infarction after coronary bypass surgery

**DOI:** 10.1186/1757-1626-2-9094

**Published:** 2009-11-26

**Authors:** Sanjay Sinha, Sreenivasa R Sirigiri, Srinivas K Kanakamedala, Manoj K Singh, Rakesh M Sharma

**Affiliations:** 1 Department of Urology, Medwin Hospital, Chirag Ali Lane, Hyderabad-500001, India; 2 Department of Neurology, Medwin Hospital, Chirag Ali Lane, Hyderabad-500001, India

## Abstract

**Introduction:**

Vascular spinal cord injury following coronary bypass grafting surgery is very rare and this is probably one of few reports of a presumptive partial conus medullaris lesion leading to sudden onset bladder and bowel incontinence which was managed using duloxetine, a selective serotonin and norepinephrine reuptake inhibitor. Duloxetine has been used in selected patients with post-prostatectomy sphincteric incontinence but not, to our knowledge, for spinal vascular lesions.

**Case presentation:**

A 63-year-old Indian man developed bladder and bowel incontinence immediately following coronary bypass grafting surgery. Findings were suggestive of microcirculatory partial conus medullaris infarction. Based on his urodynamics findings he was managed with duloxetine, tolterodine and clean intermittent catheterization. The clinical presentation, serial urodynamic findings and implications are discussed.

**Conclusion:**

Spinal injury following coronary bypass grafting is rare and devastating. It is important to be able to recognize the problem even when paraplegia is not noted, counsel the patient and manage the patient with the help of urodynamics. In patients with sphincteric incontinence, duloxetine may have a role in management.

## Introduction

Vascular spinal cord injury following coronary artery bypass grafting surgery (CABG) is very rare [1,2] and this is probably one of few reports of a presumptive conus medullaris lesion leading to sudden onset bladder and bowel incontinence. Our patient was managed using duloxetine, a selective serotonin and norepinephrine reuptake inhibitor with clean intermittent self catheterization (CISC). Duloxetine has been used for post-prostatectomy incontinence in men and is approved for female stress urinary incontinence in many countries [3]. The drug works by increasing the rhabdosphincter tone via a spinal cord mediated action [3]. To our knowledge, there is no literature on the use of duloxetine for neurogenic sphincteric male urinary incontinence.

## Case Presentation and Management

A 63y hypertensive, non-smoker male underwent CABG in July 2004. He was non-diabetic, had no prior neurological problems and had a normal lipid profile. He had mild urinary frequency prior to CABG. Immediately following CABG he developed both bladder and bowel incontinence. Ultrasonographic examination showed 150 ml urine in the bladder with inability to void suggesting a picture of overflow incontinence albeit with a reduced capacity. He had profound laxity of anal sphincter, distended rectum with stool impaction, impaired perianal sensation and absent bulbocavernosal reflex but no lower limb neurological symptoms. There was isolated bladder and bowel involvement without a well defined spinal shock. Lower limb reflexes were intact and normal. Spinal MR imaging did not show any abnormality. The clinical findings were consistent with probable microcirculatory ischemic infarction of the conus medullaris region leading to partial and selective denervation.

**Figure 1:**
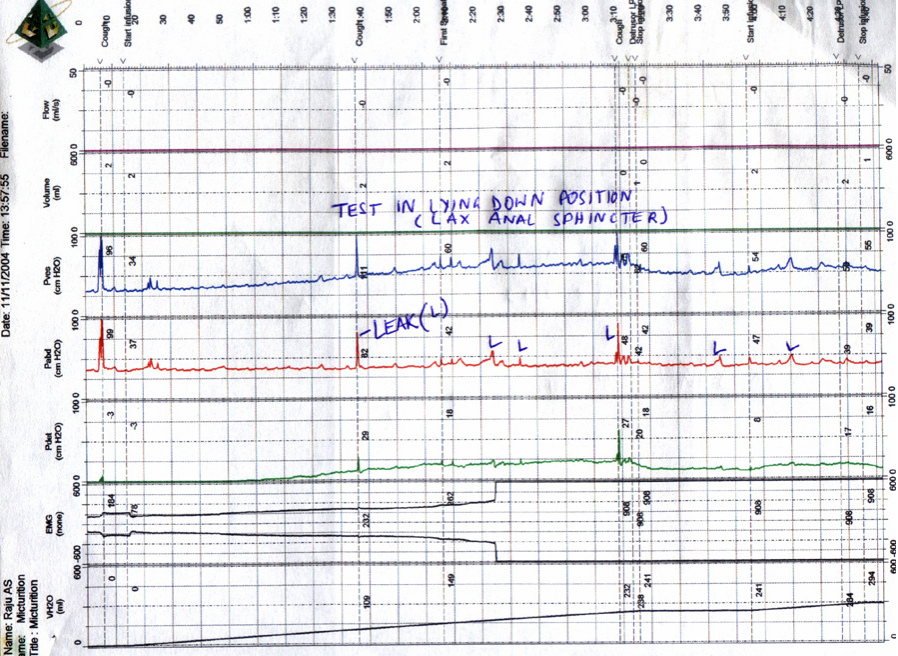
**Initial urodynamics study**. Urodynamic study prior to duloxetine (Nov 2004, 4 months after onset) done in lying down position (since the patient had a very lax anal sphincter). There was severe leak (L) noted on minimal abdominal straining. Continuous pericatheter leak was noted at 225 ml at a pressure of 19 cm H20. He had an acontractile detrusor.

**Figure 2:**
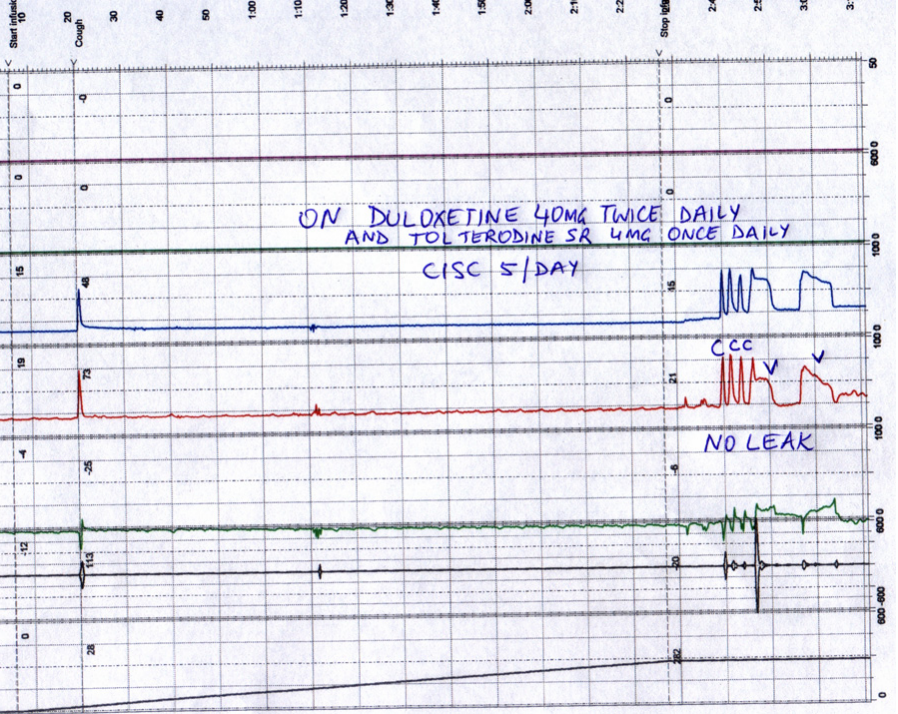
**Follow up urodynamics study**. Follow up urodynamic study after commencing 40 mg twice daily Duloxetine (Aug 2005). Patient also on tolterodine sustained release 4 mg once daily. No leak was observed on cough (C) or valsalva (V) and there was no pericatheter leak on filling beyond 400 ml (not depicted; separate cycle of filling).

He had recurrent retention for which he underwent urodynamics evaluation at 4 months. This showed mild reduction in compliance, no phasic contractions, and leak after 225 ml filling at a detrusor leak point pressure of 19 cm H20. He had severe incontinence with any rise in abdominal pressure suggesting low sphincteric resistance (Figure 1). He was unable to empty the bladder effectively on a Crede's maneuver and was started on CISC, tolterodine and imipramine. He continued to have urinary incontinence between catheterizations. He was then started on duloxetine 40 mg twice daily along with tolterodine. This resulted in complete resolution of his incontinence, increase in average CISC volumes from 150 ml to 280 ml, with marked improvement in urodynamics parameters (Figure 2).

At 5 years followup the patient continues CISC and medication and is doing well.

## Discussion

Conus medullaris ischemia and infarction with profound loss of anal sphincter tone and bowel and bladder incontinence [4] has been reported following aortic surgery due to intraoperative insult to vascular supply [5]. The clinical presentation of this patient strongly suggests the occurrence of microcirculatory partial conus medullaris infarction. Microcirculatory infarction is well known in a post operative setting and may not be identifiable on conventional imaging [2].

Duloxetine is approved for use in men for its neuropsychiatric effects [6]. Conceptually, the rhabdosphincter effects of duloxetine seen in women should be reproducible in men. Increased serotonin and norepinephrine availability due to duloxetine has been shown in healthy volunteer men at clinical dosages [7]. In fact, retention has been reported in men using the drug for psychiatric indications [6]. In our patient tolterodine was used along with duloxetine to reduce the storage bladder pressures and improve functional bladder capacity. The safety of this combination has already been documented and no dosage adjustments are necessary [8]. There are initial reports on the use of duloxetine for post-prostatectomy male urinary incontinence [9]. Duloxetine was found to be effective but there were significant side effects necessitating stoppage of drug in 6 out of the 20 patients studied. In our patient with a neurogenic male sphincteric incontinence, presumably the duloxetine worked by increasing the tone of the external rhabdosphincter by increasing neurotransmitter availability. This might have been possible because the patient had a partial conus lesion. Future studies are needed to evaluate the use of this drug in neurogenic male sphincteric incontinence to identify whether the drug would be effective in all forms of neurogenic sphincteric incontinence or only in select patients with incomplete lesions.

## Conclusion

Spinal injury following CABG is very rare. It is important to be able to recognize lower urinary tract dysfunction even when paraplegia is not noted, counsel the patient and manage the patient with the help of urodynamics. In patients with neurogenic sphincteric incontinence, duloxetine may have a role in management.

Institutional ethics committee approval was taken prior to use of duloxetine in this patient.

## Abbreviations

CABG: Coronary artery bypass grafting; CISC: Clean intermittent self catheterization.

## Consent

Written informed consent was obtained from the patient for publication of this case report and accompanying images. A copy of the written consent is available for review by the Editor-in-Chief of this journal.

## Competing interests

The authors declare that they have no competing interests.

## Authors' contributions

SS analysed and interpreted the report. SSR analysed and interpreted the report. KSK was a major contributor in writing the manuscript. MKS analysed and interpreted the report. RS was a major contributor in writing the manuscript. All authors read and approved the final manuscript.
